# Xanthomonas bundabergensis sp. nov., Xanthomonas medicagonis sp. nov. and Xanthomonas tesorieronis sp. nov.: three members of group 1 Xanthomonas

**DOI:** 10.1099/ijsem.0.006686

**Published:** 2025-03-10

**Authors:** Daniel J. E. McKnight, Johanna Wong-Bajracharya, Efenaide B. Okoh, Fridtjof Snijders, Fiona Lidbetter, John Webster, Mathew Haughton, Steven P. Djordjevic, Daniel R. Bogema, Toni A. Chapman

**Affiliations:** 1 NSW Department of Primary Industries and Regional Development, Elizabeth Macarthur Agricultural Institute, Woodbridge Rd, Menangle, NSW, Australia; 2 University of Technology Sydney, 15 Broadway, Ultimo, NSW, Australia; 3 Western Sydney University, Penrith, NSW, Australia

**Keywords:** bioinformatics, biosecurity, nonpathogenic, phylogenetics, *Xanthomonas*

## Abstract

Between 1976 and 2010, four bacterial isolates were collected in New South Wales and Queensland, Australia, and stored as part of routine biosecurity surveillance. Recently, these historic isolates were analysed as part of a larger project to enhance the taxonomic accuracy of our culture collection and improve Australia’s biosecurity preparedness. Three isolates were collected from *Fragaria × ananassa*, initially identified as *Xanthomonas* sp., and one from *Medicago sativa*, identified as *Xanthomonas axonopodis* subsp. *alfalfae*. In this study, we employed modern phenotypic and genomic techniques to further characterize these isolates. Matrix-assisted laser desorption ionization–time of flight MS biotyping and Biolog GEN III MicroPlates confirmed that they are members of the *Xanthomonas* genus but did not allow for species-level classification. Genome-relatedness indices and phylogenetic analysis confirmed that they were *Xanthomonas* and revealed that they represent three novel species. The maximum average nucleotide identity and digital DNA–DNA hybridization values observed when comparing the four isolates to all *Xanthomonas* type strains and each other were 93.9% and 50.7%, respectively. Pathogenesis assays confirmed that two of the isolates are not pathogenic to *Fragaria*, the plant from which they were isolated. Based on these findings, we propose the names *Xanthomonas bundabergensis* sp. nov. (DAR 80977^T^=ICMP 24943), *Xanthomonas medicagonis* sp. nov. (DAR 35659^T^=ICMP 24942) and *Xanthomonas tesorieronis* sp. nov. (DAR 34887^T^=ICMP 24940).

## Introduction

The genus *Xanthomonas* contains 36 validly published phytopathogenic and plant-associated bacterial species. They have a wide host range of over 400 plant species, including many economically important crops, making them a globally significant threat to agriculture. Common symptoms of *Xanthomonas* spp. infection include black chaff, black rot, blights, cankers, leaf and fruit spots and vascular wilt [1]. The genus is phylogenetically divided into two major clades, group 1 and group 2 [2]. Group 1 is a smaller clade that contains four out of the five documented non-pathogenic species, while group 2 contains the economically important plant pathogens *Xanthomonas arboricola*, *Xanthomonas campestris*, *Xanthomonas citri* and *Xanthomonas oryzae*.

While the majority are considered pathogenic, a growing number of non-pathogenic *Xanthomonas* spp. were recently discovered, including *Xanthomonas bonasiae*, *Xanthomonas maliensis*, *Xanthomonas rydalmerenesis*, *Xanthomonas sontii* and *Xanthomonas youngii* [3,6]. Additionally, non-pathogenic strains of pathogenic species have been reported, including *X. arboricola* and *Xanthomonas euroxanthea* [7,8]. These non-pathogenic strains and species garner less attention due to their minimal economic impact and the challenges associated with their detection, as they do not produce conspicuous disease symptoms. The first report of a non-pathogenic *Xanthomonas* strain isolated from healthy plant tissue was not published until 1996 [9]. However, these isolates were only reported as unclassified *Xanthomonas* sp. and were not designated a species. Despite their benign nature, it is important to identify and characterize non-pathogens for effective disease surveillance and biosecurity. Studying non-pathogenic species enhances our understanding of the genes and disease mechanisms that enable pathogenic species to infect their hosts. Additionally, plant-associated bacteria are often found living in symbiotic relationships on plants. This interaction between both closely and distantly related bacteria creates an environment conducive to horizontal gene transfer (HGT). HGT allows for the transfer of genetic content between organisms and potentially transforms non-pathogenic strains into new pathogenic lineages. Furthermore, genomic analysis of non-pathogenic species provides a deeper understanding of the phylogeny of the species and the broader genera and facilitates more informed decision-making when identifying and prioritizing emerging pathogens. As such, the study of non-pathogenic species can help us understand the mechanisms of plant disease and aid in future identification.

*Xanthomonas* species have a range of virulence-associated factors that help them infect plant hosts. These include exopolysaccharides (EPS), lipopolysaccharides (LPS), and type I to type VI secretion systems (T1SS–T6SS). Nearly all *Xanthomonas* sp. produce the EPS xanthan gum, a heteropolysaccharide composed of glucose, glucuronic acid and mannose [10]. Xanthan gum is excreted from the cell and gives *Xanthomonas* sp. their characteristic yellow mucoid colonies. EPS helps the colonies adhere to surfaces, enhance stress tolerance and form biofilms [11]. LPS is a major component of the outer membrane of Gram-negative bacteria [12]. It contributes to cell wall integrity, aids in cellular adhesion and protects against chemicals and toxins [12,14]. Secretion systems are protein complexes that translocate protein effectors, toxins and adhesins out of the cell. Depending on the secretion system, these chemicals are translocated into either the cytoplasm of a host cell, neighbouring bacterium or into the extracellular environment [15]. This aids in host colonization, outcompeting other bacteria and adhering to surfaces. The type III secretion system (T3SS) is considered a major virulence determinant in Gram-negative bacteria, particularly in *Xanthomonas* spp., as it is closely associated with pathogenicity [15]. T3SSs secrete type III effectors (T3E) that interfere with the host immune response and molecular signalling, which aids in colonization. The *in silico* detection of virulence-associated factors such as EPS, LPS and secretion systems in *Xanthomonas* species can provide valuable insights into their pathogenic potential.

The *Xanthomonas* genus was first proposed in the early twentieth century when bacterial species were originally delineated using phenetic analysis. At the time, it was assumed that *Xanthomonas* pathogens infecting different hosts or producing different symptoms were separate species [16]. This assumption led to unreliable taxonomic classifications, which were later revised using data from techniques like DNA–DNA hybridization and *gyrB* gene phylogenetic analysis [17,18]. With the increasing availability of modern techniques like whole-genome sequencing, more comprehensive genomic analysis can be used to accurately delineate species. Two techniques widely used for this purpose are multilocus phylogenetics and genome-relatedness indices. Multilocus phylogenetics infers phylogenetic trees using multiple genes from various loci, which allows accurate visualization of the evolutionary relationships between species within a genus. Analysing a phylogenetic tree that includes type strains of known species and newly sequenced isolates can reveal potential novel species. Genome-relatedness indices, such as average nucleotide identity (ANI) and digital DNA–DNA hybridization (dDDH), provide a quantitative measure of the genetic similarity between two genomes. ANI values greater than 95–96% and dDDH values greater than 70% typically indicate that two genomes belong to the same species [19,20]. Combining multilocus phylogenetics and genome-relatedness indices allows for more accurate taxonomic classification.

In this study, we identify and classify three previously undescribed species within the *Xanthomonas* genus. This was performed using biochemical characterization, matrix-assisted laser desorption ionization–time of flight MS (MALDI-TOF MS) and both genome-relatedness indices and phylogenetic analysis to delineate the new species.

## Origin and isolation

Between 1976 and 2010, four bacterial strains were isolated in New South Wales (NSW) and Queensland (QLD), Australia, as part of routine biosecurity monitoring. Three of these strains were collected from *Fragaria × ananassa,* with DAR 34887^T^ and DAR 34893 isolated in 1976 in Rockley, NSW, and DAR 80977^T^ isolated in Bundaberg, QLD, in 2010. The fourth isolate, DAR 35659^T^, was collected from *Medicago sativa* in 1981 in Tatham, NSW. The metadata for DAR 34887^T^ and DAR 34893 confirm that they were specifically collected from leaves, but that information for the other two isolates was not recorded. Initial tests identified the strains from *Fragaria × ananassa* as undetermined *Xanthomonas* spp. and the strain from *M. sativa* as *Xanthomonas axonopodis* subsp. *alfalfae*. All metadata and assembly information for the isolates can be found in Table S1 (available in the online Supplementary Material). The isolates were lyophilized and stored at 4 °C in glass ampoules under a vacuum at the NSW Plant Pathology and Mycology Herbarium as specimens DAR 34887^T^, DAR 34893, DAR 35659^T^ and DAR 80977^T^.

## Growth and recovery from culture collection

The bacterial cultures were recovered using the method described in McKnight *et al*. [5]. Colonies grew to 2–3 mm in diameter and were slightly convex, yellow and mucoid in appearance. Cells are Gram-stain-negative, aerobic and rod-shaped. All four isolates are publicly accessible at the NSW Plant Pathology and Mycology Herbarium, and the type strains were deposited in the New Zealand International Collection of Microorganisms from Plants (ICMP) with the following codes: DAR 34887^T^=ICMP 24940; DAR 35659^T^=ICMP 24942; DAR 80977^T^=ICMP 24943. These isolates can be ordered using their accession numbers from the ICMP (https://www.landcareresearch.co.nz/tools-and-resources/collections/icmp-culture-collection/depositing-and-ordering-strains/) and the NSW Plant Pathology and Mycology Herbarium (https://www.dpi.nsw.gov.au/about-us/services/collections/collection-services).

## DNA extraction, sequencing and genome assembly

We extracted the DNA of the novel isolates at the Elizabeth Macarthur Agricultural Institute. Illumina short-read sequencing and library preparation were conducted according to Bogema *et al*. 2018 [21]. Oxford Nanopore library preparation, long-read sequencing and genome assembly were performed as described in McKnight *et al*. [5].

## Phylogenetic analysis

**Fig. 1.**
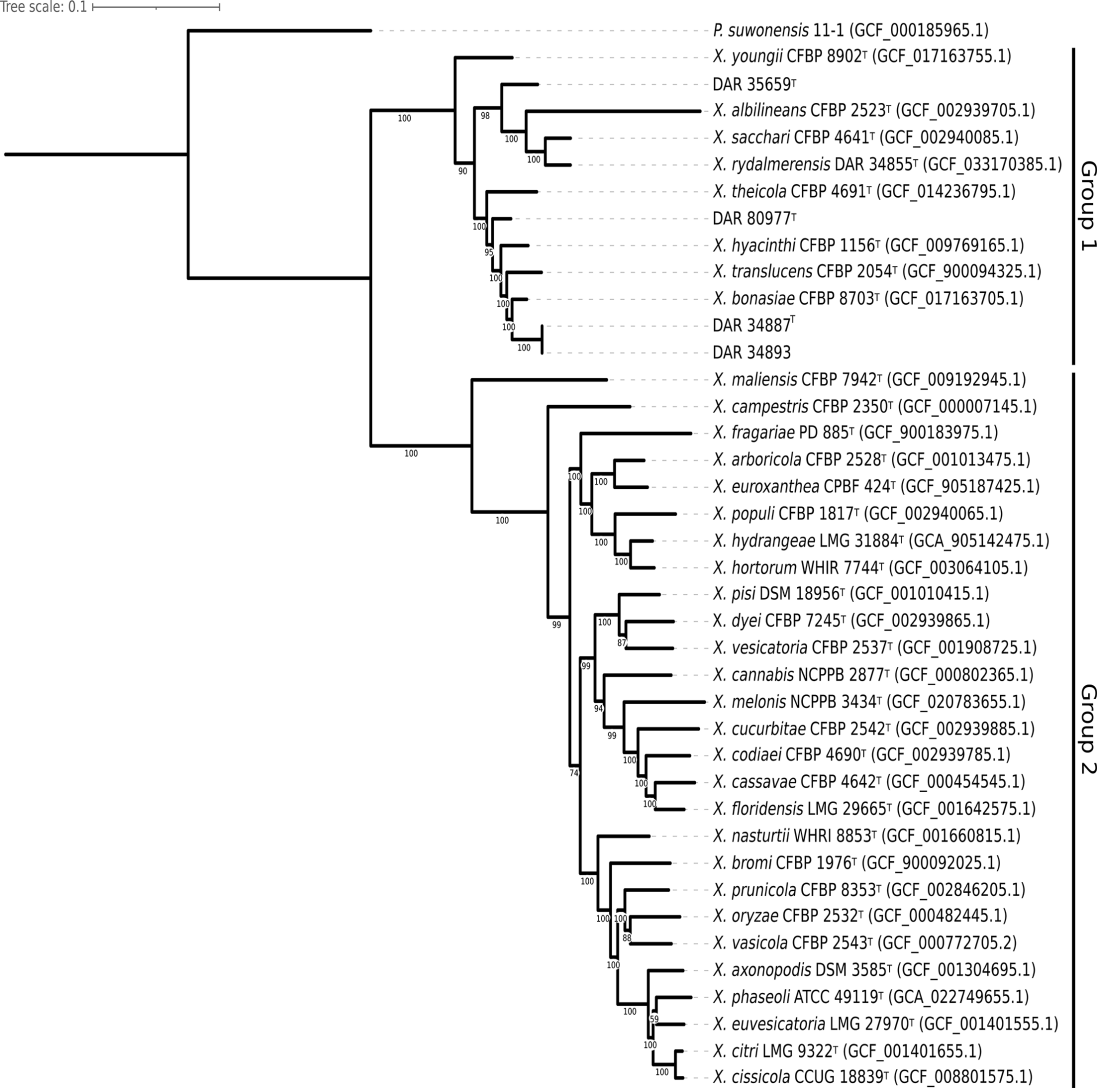
Outgroup rooted multilocus phylogenetic tree inferred using UBCG and IQ-TREE. This tree contains the novel isolates described in this study, all official *Xanthomonas* type strains and *Pseudoxanthomonas suwonensis* as an outgroup. The two major clades were labelled according to Young *et al*. [2].

Initial analysis conducted upon the collection of our isolates indicated that they are members of the *Xanthomonas* genus, with one designated as *X. axonopodis* subsp. *alfalfae* (DAR 35659^T^). To visualize the phylogenetic position of our isolates within the genus, we performed phylogenetic analysis using our isolates, all *Xanthomonas* type strains and *Pseudoxanthomonas suwonensis* as an outgroup. A common phylogenetic marker used to identify bacterial species is the 16S rRNA gene, but as shown in previous studies, the genetic variability of this gene is too low in *Xanthomonas* spp. to distinguish isolates at the species level [22]. Instead, *gyrB* is often used because it is highly conserved yet possesses enough genetic variability to differentiate species [18]. However, for increased resolution, we used the up-to-date bacterial core gene (UBCG) to extract, align and concatenate 92 single-copy genes that are common to most bacterial species. This concatenated alignment was then used to generate a maximum likelihood phylogeny using IQ-TREE v2.30 [23]. The built-in model finder chose GTR+F+R6 as the most suitable model, and branch support values were calculated using 100 non-parametric bootstrap replicates. The phylogenetic tree was then visualized and outgroup rooted using interactive Tree Of Life (iTOL) (Fig. 1) [3].

The four strains formed three single isolates or monophyletic sub-clades with branch lengths longer or consistent with several other leaves of the *Xanthomonas* species tree. All isolates sequenced here were positioned within the group 1 *Xanthomonas*, where four out of five previously documented non-pathogenic *Xanthomonas* species are found. All branch support values in group 1 were ≥90, indicating strong support for the positioning of the strains. Discrete sub-clade grouping with high bootstrap values provides significant evidence that our isolates are not members of any known *Xanthomonas* species.

## Genome-relatedness indices

Genome-relatedness indices were used to determine whether novel isolates examined here are members of a previously characterized *Xanthomonas* species. To confirm this, we used both ANI using FastANI v1.32 [24] and dDDH using the Genome-to-Genome Distance Calculator 3.0 with formula 2 [25]. We calculated ANI and dDDH through a pairwise comparison of our novel isolates with all official *Xanthomonas* type strains. ANI values greater than 95–96% and dDDH values greater than 70% were used to signify that two genomes belong to the same species [19,20]. During testing, we determined that our two isolates, DAR 34893 and DAR 34887 ^T^, were clones as they produced an ANI value of 100%. This result was reflected in the phylogeny as these isolates had identical branch lengths. As such, for all analyses in this study, we used DAR 34887 ^T^ as the type strain, as this isolate was collected first.

We performed a pairwise comparison of our isolates (DAR 34887^T^, DAR 35659^T^ and DAR 80977^T^) to all *Xanthomonas* type strains and each other. The resulting values were less than the ANI and dDDH species delineation thresholds, indicating that they do not represent a previously described *Xanthomonas* species. In each case, the closest related type strains were the group 1 *Xanthomonas* species, *X. bonasiae*, *X. hyacinthi* and *X. translucens* (Table 1). All raw data can be found in Tables S2 and S3.

**Table 1.** Pairwise ANI and dDDH results comparing novel species with the most closely related *Xanthomonas* type strains The species delineation threshold for ANI and dDDH are 95–96% and 70%, respectively.

	**ANI**			dDDH		
	***X. bonasiae***	***X. hyacinthi***	***X. translucens***	***X. bonasiae***	***X. hyacinthi***	***X. translucens***
**DAR 34887^T^**	93.9%	92.6%	92.2%	50.7%	46.5%	45.1%
**DAR 35659^T^**	90.8%	90.5%	89.9%	38.3%	39.3%	37.4%
**DAR 80977^T^**	93.9%	93.5%	92.6%	49.4%	49.9%	45.3%

In addition to comparing against official *Xanthomonas* type strains, we used ANI to conduct a comprehensive comparison of our isolates with all *Xanthomonas* assemblies in the National Center for Biotechnology Information (NCBI) GenBank database. The 7097 entries listed under the *Xanthomonas* genus as of 10 March 2024 were downloaded using the NCBI datasets command-line interface [26]. This contained all entries in both the RefSeq and GenBank databases and thus contained many duplicates, with a total of 4132 unique isolates. Using the novel isolates as the reference and NCBI isolates as the query, we used FastANI v1.32 to compare the genome pairwise. We determined that there were no isolates in the NCBI database that were above the ANI speciation threshold of 95–96%. The two closest isolates were collected in Japan (CFBP 8444 and NCPPB 2983) and produced values of ~94.2% with DAR 80977^T^. They were submitted as *X. campestris* pv. *phormiicola*; however, the NCBI taxonomy check suggested that they were most likely *X. bonasiae*, with an ANI of 94.7%.

Our three strains were found to be below the ANI and dDDH species delineation threshold when compared against *Xanthomonas* type strains and all *Xanthomonas* isolates in the NCBI database. These findings, along with the phylogenetic analysis, strongly suggest that our isolates represent three novel *Xanthomonas* species. However, while these data support this conclusion, we recognize that these novel species are represented by single isolates and a type strain with a clonal strain. This is particularly relevant for DAR 34887^T^ and DAR 80977^T^, as they are close to the species delineation thresholds when compared to *X. bonasiae*. Consequently, future data may support reclassification to a single species or species complex.

## Biochemical characterization

We performed biochemical characterization of isolates DAR 34887^T^, DAR 35659^T^ and DAR 80977^T^ using Biolog GEN III MicroPlates (Biolog) according to McKnight *et al.* [5]. We compared the data from our novel strains to the two most closely related *Xanthomonas* species. *X. bonasiae* is the most closely related species to all three novel strains, but no Biolog microplate data for this species are currently available. The results were condensed by removing tests where all strains produced the same result (Table 2). All data from the microplate assays can be found in Table S4.

**Table 2.** Biolog GEN III MicroPlate results from the novel isolates and two closely related species +, Positive, −, negative, v, variable. *X. hyacinthi* and *X. translucens* data were obtained from the Biolog GEN III database. The data presented are condensed to remove tests where all strains produced the same result.

	DAR 34887**^T^**	DAR 35659**^T^**	DAR 80977**^T^**	*X. translucens*	*X. hyacinthi*
*α*-d-Lactose	+	+	+	+	−
*α*-Hydroxy-butyric acid	−	−	−	+	−
*α*-Keto-butyric acid	v	−	v	+	−
*α*-Keto-glutaric acid	+	w	+	+	+
*β*-Hydroxy-d,l-butyric acid	−	−	−	+	+
*β*-Methyl-d-glucoside	+	+	+	−	−
*γ*-Amino-butyric acid	v	−	v	+	+
3-Methyl glucose	v	v	−	+	−
Acetoacetic acid	+	+	+	−	+
Aztreonam	+	+	+	−	+
Citric acid	v	+	+	+	+
d-Arabitol	−	−	−	+	−
Cellobiose	+	+	+	+	−
d-Fructose-6-PO4	w	+	w	+	+
d-Fucose	−	−	−	+	−
d-Galacturonic acid	+	+	w	+	+
d-Glucose-6-PO4	−	−	−	+	+
d-Glucuronic acid	−	v	−	+	+
d-Lactic acid methyl ester	v	v	v	−	−
d-Malic acid	−	v	−	+	−
Melibiose	+	+	+	+	−
Raffinose	−	v	−	−	−
d-Salicin	+	+	+	+	−
d-Serine	w	v	w	−	−
D-Turanose	+	+	+	−	−
Formic acid	v	−	v	+	−
Fusidic acid	+	+	v	+	+
Gentiobiose	+	+	+	−	−
Glucuronamide	v	w	w	+	+
Glycyl-l-proline	v	−	v	+	+
l-Alanine	v	+	v	+	−
l-Arginine	−	−	−	+	−
l-Aspartic acid	w	+	+	+	+
l-Fucose	+	+	+	+	−
l-Galactonic acid lactone	w	v	v	−	−
l-Histidine	v	−	v	+	−
l-Lactic acid	+	+	+	+	−
l-Rhamnose	−	v	−	−	−
l-Serine	v	v	v	+	+
Methyl pyruvate	w	+	+	+	+
Mucic acid	−	−	−	+	−
*myo*-Inositol	−	−	−	+	−
*N*-Acetyl-d-galactosamine	+	+	−	−	−
*N*-Acetyl-*β*-d-mannosamine	v	v	v	−	−
Nalidixic acid	−	−	−	−	+
Niaproof 4	−	+	−	−	−
pH 5	−	−	−	+	−
Propionic acid	+	+	+	+	−
Quinic acid	−	−	+	−	−
Rifamycin SV	+	v	v	+	+
Tetrazolium blue	+	+	+	−	+
Tetrazolium violet	+	+	+	−	−
Vancomycin	−	+	v	−	−

Both *X. translucens* and *X. hyacinthi* can be differentiated from other strains by their ability or inability to utilize certain substrates (d-arabitol, d-fucose, acetoacetic acid, d-cellobiose, d-melibiose and d-salicin). DAR 35659^T^ can be distinguished by its resistance to the surfactant Niaproof 4 and antibiotic vancomycin, while DAR 80977^T^ can be separated by its utilization of quinic acid. DAR 34887^T^ could potentially be distinguished by its weak utilization of l-aspartic acid and methyl pyruvate, which the other strains can strongly utilize.

## MALDI-TOF MS analysis

For future identification of these species, spectra were generated using a Bruker MALDI-TOF Biotyper Microflex LT (Bruker Daltonics). The MALDI-TOF MS analysis was performed as per McKnight *et al*. [5], and the MALDI BioTyper Main Spectra (BTMSP) files are available at https://doi.org/10.6084/m9.figshare.26047660. Log scores produced from the analysis were interpreted as follows: >2.3 indicated highly probable species match, 2.0–2.3 as secure genus and probable species identification, 1.7–2.0 as probable genus identification and <1.7 as unreliable identification [27,28].

The highest score for DAR 34887^T^ and DAR 34893 was each other with a value of 2.760, which was expected as they are clonal. This was followed by *X. bonasiae* and *Xanthomonas rydalmerensis* isolates with log scores ranging from 1.730 to 2.120, indicating secure genus identification and probable species identification. This result is expected as these species group closely in phylogenetic analysis and produce ANI values of 93.9% with *X. bonasiae*. The highest log score for DAR 35659^T^ is also from our *X. rydalmerensis* isolates with 1.820, indicating probable genus identification. Lastly, DAR 80977^T^ produced a log score of 1.770 with an * X. hyacinthi* isolate, again indicating genus-level identification. As such, DAR 35659^T^ and DAR 80977^T^ can be accurately detected using MALDI-TOF MS; however, *Xanthomonas tesorieronis* may be mistaken for *X. bonasiae* if databases are not updated with the type strain DAR 34887^T^.

## Presence and absence of virulence-associated factors

**Fig. 2.**
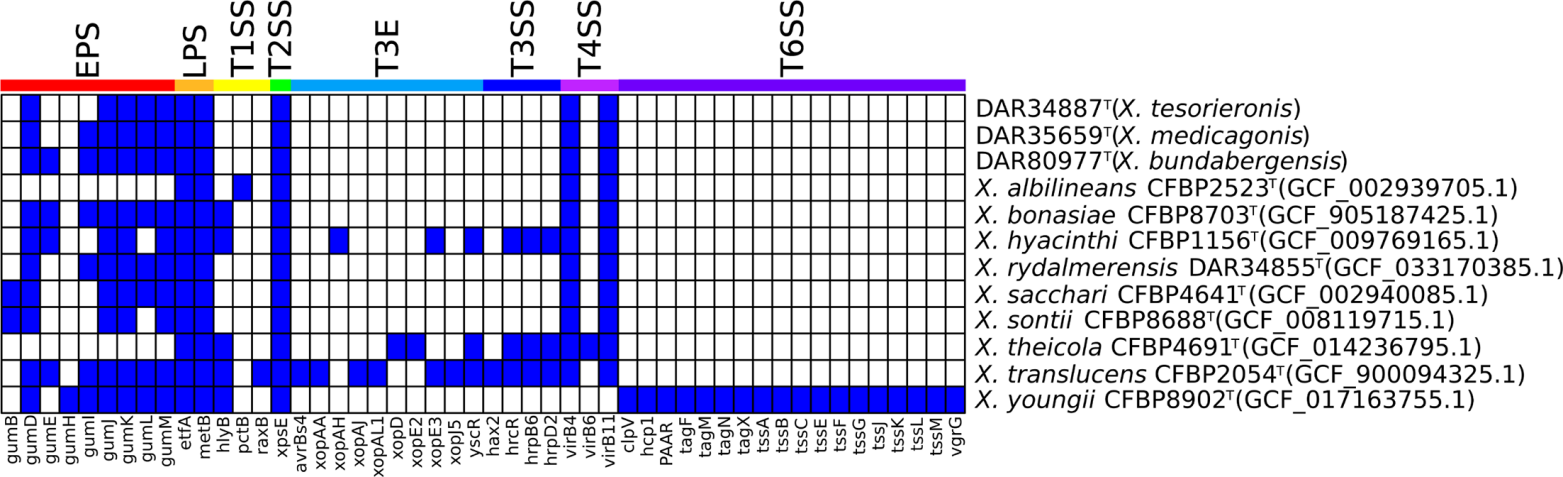
The presence/absence results of virulence-associated factors in novel species and group 1 *Xanthomonas* type strains. Blue and white signify the presence and absence of a gene, respectively, with ≥70% length similarity, ≥70% alignment similarity and an *e*-value ≤1e^−10^. The coloured bars and labels at the top of the figure represent what virulence system each gene belongs to. Type strains are denoted as species name, strain name and RefSeq accession number. The data presented have been condensed to only show genes that were present in at least one isolate.

To investigate the presence of *Xanthomonas*-specific virulence-associated factors in the genomes of the three novel species, we performed a blastx search using Diamond v2.0.15 [29]. We set no limit on the maximum number of target sequences and filtered for results that were ≥70% length similarity, ≥70% alignment similarity and an *e*-value ≤1e^−10^. To provide context for our findings, we performed a comparative analysis by including the type strains of all group 1 *Xanthomonas* species. Genomes were kept as nucleotide sequences and were compared against our reference database of known *Xanthomonas* virulence-associated factors. This included EPS, LPS marker genes, T3E, T3SS, type IV secretion system (T4SS) and T6SS genes from *Getaz et al.* [30]. We additionally included proxy genes to detect the presence of T1SS, type II secretion system (T2SS) and type V secretion system (T5SS) acquired from *Alvarez-Martinez et al.* [15]. Additional EPS genes were extracted from an *X. oryzae* pv. *oryzae* genome (AP008229.1) to obtain all genes of the gum operon. The accession numbers for all genes used in the study are listed in Table S5. The presence/absence data were visualized using R v4.3.0 [31] with the R package Pheatmap v1.0.12 [32] (Fig. 2).

Analysis showed that all isolates possess very few virulence-associated factors compared to those of known pathogenic *Xanthomonas* species. All type strains and our novel isolates contained 5–7 EPS genes, except *Xanthomonas albilineans* and *Xanthomonas theicola,* where no EPS genes from our database were detected. All isolates in the analysis were shown to possess the genes etfA and metB which indicate the location of the LPS gene cluster. The coordinates of these genes were used to manually extract and align the LPS gene cluster in our novel species to study their composition. This analysis revealed that each novel species possesses a distinct LPS cluster, with 12–20 LPS-associated proteins. These LPS and EPS genes encode polysaccharides that aid in forming biofilms, cellular adhesion and stress tolerance.

None of the three T1SS proxy genes were detected in the analysis, besides *X. albilineans*, which was found to possess *HlyB*. Detection of this gene signals the potential presence of the *hlyDB* gene cluster. Two proxy genes were used to detect the presence of the T2SS, with *xps* generally conserved across the *Xanthomonas* genus and *xcs* found in a select few with unknown functions [15]. The *xps* proxy gene was found in all group 1 *Xanthomonas* species in our analysis, signalling the presence of the *xps* gene cluster that expresses the T2SS. Due to its ubiquity between both confirmed non-pathogens (*X. bonasiae*, *X. youngii, X. rydalmerensis* and *X. sontii*) and pathogenic species, it is unlikely that it is a major contributor to pathogenesis in these species. None of the four T5SS proxy genes in our database were detected in any of the type strains.

The T3SS is a major virulent determinant in pathogenic species, but the largest repertoire in our analysis was in *X. translucens*, a known pathogen of major cereal crops, with six T3E and five T3SS genes. With so few genes, the T3SS would not be functional; however, it may utilize alternate virulence factors for infection. All but two type strains in the analysis contained the same two T4SS genes (*virB4* and *virB11*) of the eight genes in our database. The exceptions were *X. youngii* with no T4SS genes and * X. theicola* that additionally contained *virB6*. With so few T4SS genes, it is unlikely that any of these species can express a functional T4SS. The only species in the analysis that was found to possess T6SS genes was *X. youngii*, containing 18 of the 19 T6SS genes in our reference database. It was shown to have all genes required for the expression of the T6SS membrane-spanning complex, baseplate and extended inner tube with contractile sheath [15]. This indicates a high probability that this strain has a functioning T6SS; however, a pathogenicity study demonstrated that *X. youngii* was non-pathogenic to the plant from which it was collected [3]. However, it did produce hypersensitive reactions in geranium, melon, squash, tobacco and tomato leaves after infiltration. The role of the T6SS is mostly associated with anti-amoeba and antagonistic interbacterial activity [15]. However, studies of the closely related *Pseudomonas* genus have found that the T6SS can contribute to host subversion, aiding in the colonization of host plants.

Our novel isolates were found to possess only a limited number of virulence-associated factors, lacking T1SS, T3SS, T5SS and T6SS. They did, however, contain a typical number of EPS and LPS, along with proxy genes indicating the potential presence of the T2SS. These findings indicate that they have low virulence potential, which we further confirmed using a pathogenicity assay.

## Pathogenicity tests

Results from the presence/absence of virulence-associated factor analysis indicated that the three novel strains lack the mechanisms required to cause disease. We conducted a pathogenesis trial by inoculating the leaves of strawberry cv. elsanta plantlets with all four isolates as per McKnight *et al*. [5]. The trial ran for 8 weeks with stems and leaves checked for signs of infection twice weekly, but no disease symptoms or hypersensitivity was observed. These studies indicate that DAR 34887^T^, DAR 34893 and DAR 80977^T^ are not pathogenic to strawberry plants from which they were isolated. However, as DAR 35659^T^ was collected from alfalfa, its pathogenic potential is unconfirmed.

## Genome features

The genome features of each isolate were analysed using Quast v5.1.0rc1 [33] and Bakta v1.9.3 with database v. 5.1 [34] (Table 3). All four isolates had complete circular chromosomes with no plasmids. The clonal isolates, DAR 34887^T^ and DAR 34893, produced almost identical results, only differing by 2 bp in length. All isolates had guanine–cytosine (GC) content typical of *Xanthomonas* species, ranging from 68.3 to 69.5%.

**Table 3.** Genome features of the four novel isolates, generated using Quast and Bakta

	DAR 34887**^T^**	DAR 34893	DAR 35659**^T^**	DAR 80977**^T^**
**Length (bp)**	5337149	5337147	5415250	5482839
**G+C content (mol%)**	68.3	68.3	69.0	69.5
**Genome coverage**	60×	106×	140×	99×
**Coding density (%)**	87.0	87.0	86.5	87.4
**Coding Sequences**	4362	4365	4460	4483
**Transfer RNA**	57	57	55	57
**Transfer-messenger RNA**	1	1	1	1
**Ribosomal RNA**	6	6	6	6
**Noncoding RNA**	26	26	18	10
**Pseudogenes**	14	14	22	7
**Hypothetical genes**	241	244	368	221

## Description of *Xanthomonas bundabergensis* sp. nov.

*Xanthomonas bundabergensis* (bun.da.berg.en’sis. N.L. fem. adj. *bundabergensis*, pertaining to Bundaberg, a city in Australia where it was first isolated).

Colonies grew on yeast dextrose carbonate (YDC) solid agar when incubated at 25 °C for 48 h. The colonies were convex, mucoid and yellow-pigmented and grew to 2–3 mm in diameter. Cells are Gram-stain-negative, aerobic and rod-shaped. It can utilize *α*-d-glucose, *α*-keto-glutaric acid, *α*-d-lactose, *β*-methyl-d-glucoside, acetic acid, acetoacetic acid, bromo-succinic acid, citric acid, d-cellobiose, d-fructose, d-galactose, d-maltose, d-mannose, d-melibiose, d-salicin, d-trehalose, d-turanose, dextrin, gelatin, gentiobiose, glycerol, l-aspartic acid, l-fucose, l-glutamic acid, l-lactic acid, l-malic acid, methyl pyruvate, *N*-acetyl-d-glucosamine, pectin, propionic acid, quinic acid, sucrose and Tween 40. It can grow in the presence of 1% NaCl, 1% sodium lactate, aztreonam, lincomycin, pH 6, tetrazolium blue and tetrazolium violet.

This species includes the type strain DAR 80977^T^=ICMP 24943 (NCBI accession number GCF_041240605.1). Its genome is a complete circular chromosome with no plasmids, a G+C content of 69.5 mol% and a length of 5.48 Mbp. The isolate was collected from *Fragaria × ananassa* in 2010 in Bundaberg, Australia.

## Description of *Xanthomonas medicagonis* sp. nov.

*Xanthomonas medicagonis* (me.di.ca.go’nis. N.L. gen. n. *medicagonis*, of *Medicago*).

Colonies grew on yeast dextrose carbonate (YDC) solid agar when incubated at 25 °C for 48 h. The colonies were convex, mucoid and yellow-pigmented and grew to 2–3 mm in diameter. Cells are Gram-stain-negative, aerobic and rod-shaped. It can utilize *α*-d-glucose, *α*-d-lactose, *β*-methyl-d-glucoside, acetic acid, acetoacetic acid, bromo-succinic acid, citric acid, d-cellobiose, dextrin, d-fructose, d-fructose-6-PO4, d-galactose, d-galacturonic acid, d-maltose, d-mannose, d-melibiose, d-salicin, d-trehalose, d-turanose, gelatin, gentiobiose, glycerol, l-alanine, l-aspartic acid, l-fucose, l-glutamic acid, l-lactic acid, l-malic acid, methyl pyruvate, *N*-acetyl-d-galactosamine, *N*-acetyl-d-glucosamine, pectin, propionic acid, sucrose and Tween 40. It can grow in the presence of 1% NaCl, 1% sodium lactate, aztreonam, fusidic acid, lincomycin, Niaproof 4, pH 6, tetrazolium blue, tetrazolium violet and vancomycin.

This species includes the type strain DAR 35659^T^=ICMP 24942 (GCF_041242975.1). Its genome is a complete circular chromosome with no plasmids, a G+C content of 69.0 mol% and a length of 5.42 Mbp. The isolate was collected from *M. sativa* in 1981 in Tatham, Australia.

## Description of *Xanthomonas tesorieronis* sp. nov.

*Xanthomonas tesorieronis* (te.so.ri.e.ro’nis. N.L. gen. n. *tesorieronis*, named in honour of Leonard Tesoriero, a plant pathologist who made significant contributions to the projects that collected these isolates as well as general plant pathology research).

Strains of this species grow into colonies on YDC solid agar when incubated at 25 °C for 48 h. The colonies were convex, mucoid and yellow-pigmented and grew to 2–3 mm in diameter. Cells are Gram-stain-negative, aerobic and rod-shaped. It can utilize *α*-d-glucose, *α*-keto-glutaric acid, *α*-d-lactose, *β*-methyl-d-glucoside, acetic acid, acetoacetic acid, bromo-succinic acid, d-cellobiose, dextrin, d-fructose, d-galactose, d-galacturonic acid, d-maltose, d-mannose, d-melibiose, d-salicin, d-trehalose, d-turanose, gelatin, gentiobiose, glycerol, l-fucose, l-glutamic acid, l-lactic acid, l-malic acid, *N*-acetyl-d-galactosamine, *N*-acetyl-d-glucosamine, pectin, propionic acid, sucrose and Tween 40. It can grow in the presence of NaCl 1%, sodium lactate, aztreonam, fusidic acid, lincomycin, pH 6, rifamycin SV, tetrazolium blue and tetrazolium violet.

This species includes the type strain DAR 34887^T^=ICMP 24940 (GCF_041245805.1) and DAR 34893 (GCF_041245535.1). The type strain genome is a complete circular chromosome with no plasmids, a G+C content of 68.3 mol% and a length of 5.33 Mbp. The two strains were collected from *Fragaria × ananassa* in 1976 in Rockley, Australia.

## Protologue

Accession numbers for raw reads are available in the GenBank database.

**Table:** 

Isolate code	Read accession	Sequencing type
DAR 34887	SRR29429473	Illumina
DAR 34887	SRR29429472	Oxford Nanopore
DAR 34893	SRR29429471	Illumina
DAR 34893	SRR29429470	Oxford Nanopore
DAR 35659	SRR29429469	Illumina
DAR 35659	SRR29429468	Oxford Nanopore
DAR 80977	SRR29429467	Illumina
DAR 80977	SRR29429466	Oxford Nanopore

Accession numbers for assembled genomes available in the GenBank database.

**Table:** 

Isolate code	Biosample accession	Genome accession
DAR 34887	SAMN41704773	CP162490
DAR 34893	SAMN41704774	CP162489
DAR 35659	SAMN41704775	CP162488
DAR 80977	SAMN41704776	CP162487

Accession numbers for 16S rRNA gene are available in the GenBank database.

**Table:** 

Isolate code	Accession no.
DAR 34887	PQ218803.1
DAR 34893	PQ218804.1
DAR 35659	PQ218805.1
DAR 80977	PQ218806.1

## supplementary material

10.1099/ijsem.0.006686 Uncited Supplementary Material 1.

## Abbreviations

ANI: Average Nucleotide Identity
dDDH: digital DNA–DNA hybridization
EPS: exopolysaccharides
HGT: horizontal gene transfer
ICMP: International Collection of Microorganisms from Plants
LPS: lipopolysaccharides
MALDI-TOF MS: matrix-assisted laser desorption ionization–time of flight MS
NCBI: National Center for Biotechnology Information
NSW: New South Wales
QLD: Queensland
T3E: type III effectors
T1SS: type I secretion systems
T2SS: type II secretion system
T3SS: type III secretion system
T4SS: type IV secretion system
T5SS: type V secretion system
T6SS: type VI secretion systems
UBCG: up-to-date bacterial core gene
YDC: yeast dextrose carbonate
